# Effectiveness and safety of lactobacilli in children with functional constipation: Study protocol for a meta-analysis and systematic review

**DOI:** 10.1097/MD.0000000000015675

**Published:** 2019-05-17

**Authors:** Wenhao Yang, Tao He, Weijian Zhang, Li Gu, Renyuan Tu, Hanmin Liu

**Affiliations:** a Department of Pediatrics, West China Second University Hospital, Sichuan University, Guoxuexiang, Chengdu; b Key Laboratory of Birth Defects and Related Diseases of Women and Children (Sichuan University), Ministry of Education, Chengdu, Sichuan; c Department of Breast Surgery, West China Hospital/West China School of Medicine, Sichuan University, Guoxuexiang, Chengdu, China.

**Keywords:** children, constipation, lactobacilli, meta-analysis

## Abstract

**Background:**

Constipation is one of the most common diseases in children and it is also a significant healthcare burden, more than many other common childhood diseases. For some children, 1st-line treatment cannot relieve their constipation and their constipation symptoms maybe continue to adolescence. So, alternative treatment options such as lactobacilli are needed. However, the effectiveness and safety of lactobacilli is still unclear. To investigate this question, we conduct a systematic review and meta-analysis.

**Methods:**

The protocol followed Preferred Reporting Items for Systematic Reviews and Meta-Analyses Protocols. Three main databases (PubMed, Embase, and the Cochrane Library) will be searched to December 20, 2018 for randomized controlled trials investigating the effects and safety of lactobacilli for constipation in children with no language restrictions. In addition, a manual search of the references of relevant published studies will also be considered.

Two independent reviewers will conduct studies selection, data extraction, and risk of bias assessment. The primary outcome is defecation frequency, treatment success (bowl movement >3 times per week). The 2nd outcome is stool consistency, incidence of abdominal pain, patients using laxatives, and adverse events.

**Results:**

The results will provide useful information about the effect and safety of lactobacilli for constipation in children.

**Conclusion:**

The findings of this study will be published in a peer-reviewed journal.

**PROSPERO registration number:**

CRD42019125913

## 1 Introduction

Constipation is one of the most common diseases in children, with a incidence of 7% to 30% in most countries.^[1–3]^ It is also a significant healthcare burden, more than many other common childhood diseases.^[4]^ About 0.3% and 8% of the children were diagnosed in the children's admission center, while 25% of the children were diagnosed in the gastrointestinal clinic, and most of the patients were still only functional constipation.^[5]^

At present, the diagnostic standard for constipation in children mainly adopts the Rome III diagnostic standard for functional gastroenteropathy formulated in 2006.^[6]^ According to ESPGHAN and NASPGHAN guidelines, the current 1st-line treatment for constipation in infants and children is to relieve fecal obstruction and maintain treatment. However, the administration of polyethylene glycol (a laxative) decreased the relative abundance of Peptococcaceae, Eubacteriaceae, and Anaeroplasmataceae.^[7]^ In these patients, more than 40% of the 1st-line treatment is ineffective, and 30% of constipation symptoms continue to adolescence, and even some adverse reactions, such as defecation and abdominal pain, will appear after the 1st-line treatment.

Therefore, in recent years, in addition to the 1st-line treatment, alternative treatment options such as probiotics are gradually emerging. For children with functional constipation, probiotics regulate the balance of intestinal flora by producing lactic acid and short-chain amino acids,^[8]^ changing the pH value of feces and promoting intestinal peristalsis.^[9,10]^

However, especially for children, the efficacy and adverse reactions of lactobacilli in improving symptoms of constipation still remain controversial in existing randomized controlled clinical studies.^[11–13]^ Furthermore, there is no systematic review and meta-analysis to evaluate the efficacy and safety of various lactobacilli in children with functional constipation. In this study, we are aiming to perform a systematic review and meta-analysis to evaluate the efficacy and safety of various lactobacilli in the treatment of constipation in children by integrating existing randomized controlled studies.

## 2 Methods

### 2.1 Registration

This study protocol has been registered in the PROSPERO and the registration number is CRD42019125913. The *Cochrane Handbook for Systematic Reviews of Interventions* will be used as a guideline^[14]^ and the software RevMan 5.3 will be used to construct the meta-analysis. We will report this study in accordance with the PRISMA statement also.^[15]^ No ethical statement will be required for this study because there is no direct involvement of human.

### 2.2 Eligibility criteria

The eligibility criteria are summarized using PICOS approach (patients, intervention, comparisons, outcome, and study design type).

#### 2.2.1 Types of participants

Participants aged between 6 months and 18 years old will be included. All participants diagnosed with functional constipation according to the Roma III diagnostic criteria and clinical symptoms. There are no restrictions in age, ethnic distribution, and gender.

#### 2.2.2 Interventions and comparisons

The treatment group will receive lactobacilli therapy for more than 4 weeks. The control group will receive placebo for same time. Those trials reported their participants receive other probiotics or antibiotics will be excluded.

#### 2.2.3 Outcome measures

The primary outcome is defecation frequency, treatment success (bowl movement >3 times per week). The 2nd outcome is stool consistency, incidence of abdominal pain, patients using laxatives, and adverse events.

#### 2.2.4 Types of studies

Randomized controlled trials (RCTs) published with no language restriction up to December 20, 2018 will be included.

### 2.3 Search methods

PubMed, Embase, and the Cochrane Library will be systematically searched for eligible studies to December 20, 2018. The search strategy will involve terms including child, constipation, lactobacilli, and RCT. A detailed search strategy in PubMed, Embase, and the Cochrane Library is described in Table 1. Relevant studies and systematic reviews will also be scanned for additional eligible trials.

**Table 1:**
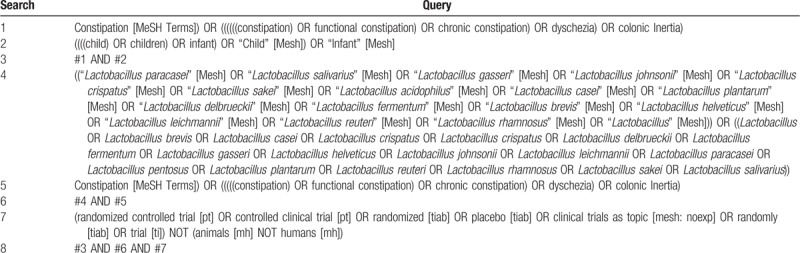
Preliminary search strategy in PubMed.

### 2.4 Study selection and data extraction

#### 2.4.1 Study selection

**Figure 1:**
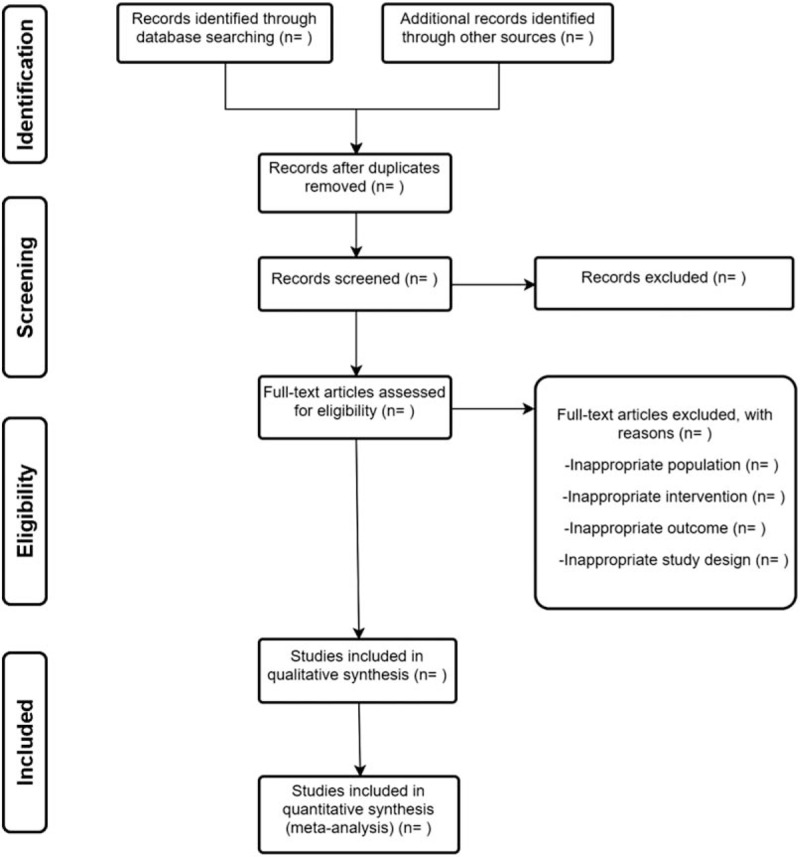
Flow diagram of study selection.

Study selection will be performed by 2 reviewers independently. The search results from 3 electronic databases and additional trials from other resources will be sent to endnote. After duplicates removed, firstly we will read the title and abstract to exclude most of trials. Secondly, full texts will be read for further exclusion. The selection process will be summarized in a PRISMA flow diagram (Fig. 1). Any disagreements between 2 authors should be resolved with the help of a 3rd author.

#### 2.4.2 Data extraction

Two reviewers will select the included studies and will extract relevant data independently from the studies. The data will include: study characteristics, patient characteristics, data needed for quality assessment, and outcomes. Patient characteristics includes: type of interventions received, mean age, sex, sample, and diagnosis criteria. Outcomes include defecation frequency, treatment success (bowl movement >3 times per week), stool consistency, abdominal pain, patients using laxatives, adverse events, and so on. All study characteristics will be summarized in the same standardized collection form. When extraction finished, data will be checked with each other by the 2 reviewers. Any discrepancies should be resolved by negotiation between the 2 reviewers with the help of a 3rd author.

### 2.5 Risk of bias assessment

Two authors will assess the methodologic quality of all included studies independently based on the Cochrane Collaboration's tool.^[16]^ The following contents will be evaluated: random sequence generation, allocation concealment, blinding of participants and personnel, blinding of outcome assessment, incomplete outcome data, selective reporting, and other biases. Each domain will be judged by the level of risk of bias: high level, low level, or unclear level. Any disagreements will be solved by discussion or with the help of a 3rd author.

### 2.6 Data synthesis and statistical analysis

#### 2.6.1 Data synthesis

The RevMan 5.3 software will be used to construct the mata-analysis. Dichotomous data, such as treatment success, painful defecation, and patients using laxatives, will be reported as risk ratios with their 95% confidence intervals (CIs). The mean difference and the 95% CI will be calculated for the continuous variable. *P* < .05 will be considered to be statistically significant.

#### 2.6.2 Assessment of heterogeneity

Heterogeneity will be assessed by the Chi-squared test and the *I*^2^ test. If *P* > .10 and *I*^2^ < 50%, the heterogeneity is acceptable and a fixed effect model will be used for data analysis. If *P* < .10 and *I*^2^ ≥ 50%, we will search for the reasons for the high heterogeneity and use a random effects model for data analysis.

#### 2.6.3 Sensitivity analysis

Sensitivity analysis will be carried out based on the sample size, the missing data result and the methodologic quality of the included study. If necessary, we will exclude a low-quality study and repeat the meta-analysis to test the stability of the pooled results.

### 2.7 Assessment of reporting bias

If more than 10 studied are included, a Begg funnel plot Egger regression will be used to examine the reporting bias. The results will be calcified based on the *Cochrane Handbook for Systematic Reviews of Interventions*.

### 2.8 Confidence in cumulative evidence

The quality of evidence will be assessed based on the Grading of Recommendations Assessment, Development, and Evaluation (GRADE) system. The evidence will be adjusted to 4 levels: high, moderate, low, or very low.

## 3 Discussion

Currently, there is still controversy over whether to use probiotics after the ineffective standard treatment for children with constipation. Some published systematic review and meta-analysis show the different effects of various probiotics in adults or children.^[17–20]^ So, it is hard to judge which probiotics plays a big role in improving the symptoms of constipation. And lactobacilli is an important component of probiotics. There are a lot of research about whether lactobacillus can improve constipation symptoms, but the result is still controversial, and there is no meta-analysis to discuss the curative effect and safety of only lactobacilli for constipation in children. Therefore, it is very necessary to conduct a systematic review and meta-analysis to investigate the effects and safety of lactobacilli for constipation in children. We aim to summarize direct evidence about published RCTs and provide evidence-based suggestions for the clinical use of lactobacilli.

## Author contributions

WHY put forward the concept of this study. WHY drafted the preliminary version of this protocol. TH and RYT will contribute to the study search, study selection, data extraction, and risk of bias assessment. WJZ and LG will complete the data analysis. WHY and HML will help to solve any disagreement and ensure the quality of this study. All authors critically reviewed, revised, and approved the final manuscript.

**Conceptualization:** Wenhao Yang.

**Data curation:** Tao He, Renyuan Tu.

**Methodology:** Weijian Zhang, Li Gu.

**Project administration:** Wenhao Yang.

**Supervision:** Hanmin Liu.

**Writing – original draft:** Wenhao Yang.

**Writing – review & editing:** Wenhao Yang, Hanmin Liu.
